# Early recognition of post-stroke spasticity: the I-REFER study

**DOI:** 10.3389/fneur.2026.1735793

**Published:** 2026-05-13

**Authors:** Klemens Fheodoroff, Benjamin Waeschle, Manuela Schuetzer, Georg Comes, Joerg Wissel

**Affiliations:** 1 KABEG Gailtal-Klinik, Neurorehabilitation, Hermagor, Austria; 2 Merz Therapeutics GmbH, Frankfurt, Germany; 3 Merz Pharma Austria GmbH, Vienna, Austria; 4 Neurology and Psychosomatics at Wittenbergplatz, Out-Patient Clinic, Berlin, Germany; 5 University of Potsdam, Health-Campus Brandenburg, Potsdam, Germany

**Keywords:** botulinum neurotoxin type A [MeSH term], decision tree [MeSH term], early recognition, muscle spasticity [MeSH term], nurses, post-stroke spasticity, stroke [MeSH term], therapists

## Abstract

**Background:**

Effective post-stroke spasticity (PSS) management relies on early recognition and treatment, but access to specialist physicians is limited and PSS remains underdiagnosed and undertreated. Empowering non-specialist nurses and therapists to identify potential harmful PSS may expedite appropriate referral. We assessed validity and inter-rater reliability (IRR) of the spastic movement disorders decision tree for upper extremity spasticity by comparing referral decisions made by nurses and therapists to those made by specialist physicians (https://clinicaltrials.gov/study/NCT06381999).

**Methods:**

The decision tree evaluates three cardinal symptoms of PSS at the elbow, wrist, and fingers using simplified versions of standard scales. Nurses and physio−/occupational therapists used the decision tree to rate each symptom as “no/minimal,” “moderate,” or “severe,” resulting in a dichotomous outcome of referral/no-referral to a specialist. Referral decisions (*n* = 69) by five nurses (primary objective) and four therapists (secondary objective) were compared with standard specialist physician decisions (two physicians).

**Results:**

IRR was high between nurses and physicians on the need for specialist referral, overall (85.5%) and at the elbow (88.4%), wrist (85.5%), and fingers (84.1%), with 84.6% sensitivity (to correctly identify patients requiring specialist referral) and 86.7% specificity (to identify those not requiring specialist referral). Positive and negative predictive values were also high overall (89.2 and 81.3%, respectively) and at all (movement) segments. Findings were similar for therapists versus physicians. Sensitivity analyses based on a categorical endpoint (low risk for PSS, assess botulinum toxin type A [BoNT-A] indication, indication for BoNT-A) showed similar trends, although IRR versus physicians was lower for both nurses and therapists compared with dichotomous results.

**Conclusion:**

This decision tree for spasticity in the upper extremities showed high validity and reliability for effectively helping nurses and therapists recognize potential PSS and initiate specialist referral for timely diagnosis and potential BoNT-A treatment. It is recommended that this tool be introduced into standard clinical practice and its potential for broader use in community settings be examined.

## 1 Introduction

Stroke is a leading cause of disability and death, with global stroke mortality estimated to increase from 6.6 million in 2020 to 9.7 million in 2050 (1). Up to 43% of stroke survivors with paresis will develop post-stroke spasticity (PSS) and motor impairments, with possible complications such as pain and contractures in the relevant (movement) segments, significantly impacting their daily activities and quality of life (2, 3). Onset of PSS can occur in both the upper and lower limbs and extremities, though with typically higher prevalence in upper limbs, such as the elbow and wrist (4). As well as the physical and mental health impact on the patient, PSS is associated with a substantial caregiver burden and wider economic burden due to reduced ability to work and increased healthcare costs (5–9).

A multimodal therapeutic approach, based on pharmacologic and non-pharmacologic strategies, is crucial to enhance the overall treatment effect across PSS-associated impairments (10–13). Non-pharmacologic interventions include both focal and generalized approaches, such as stretching, electrical stimulation, and posture or physical management strategies (11). Intra-muscular injection of botulinum neurotoxin type A (BoNT-A) is the preferred pharmacologic treatment for focal, multifocal, and segmental disabling spasticity (10–15) and works by blocking acetylcholine release at the neuromuscular junction (15, 16).

Regardless of the approach taken, effective management of spasticity ultimately relies on early recognition and treatment, and delays can result in substantial disability (10–13). BoNT-A injections are strongly recommended within 3 months following a stroke in patients with confirmed or suspected PSS, and the clinical effects of BoNT-A typically persist for up to 6 months after injection (10, 12, 17). Initiating BoNT-A treatment within 3 months following a stroke (for patients with PSS) has shown clinical benefits and improved long-term outcomes, in terms of both symptom control and avoiding complications (10, 18). However, PSS is often either diagnosed late or remains undetected, resulting in delayed or no treatment, and access to specialists experienced in identifying arm spasticity – the gold standard for assessment of PSS in the arm – (14, 19) is limited in outpatient or community care (6, 20). Claims data from 7,947 patients with PSS in Germany found that only 1% of eligible patients received BoNT-A (20), and two retrospective studies of general practitioners in Germany reported that <10% of patients with spastic movement disorders (SMDs) received BoNT-A (21, 22). Claims data also report low use of BoNT-A in patients with PSS in the United States (7% of patients who received pharmacologic treatment) (9).

To address this unmet need, it is crucial for individuals with potential PSS symptoms to be assessed by an experienced spasticity specialist to obtain a timely PSS diagnosis and access to treatment with BoNT-A (6). However, given the limited access to such physicians, there is an opportunity to empower non-specialist healthcare providers to reliably identify potential PSS and expedite appropriate referral to a specialist (13). The obvious non-specialist candidates to be trained in the identification of PSS are therapists, including physiotherapists and occupational therapists, as they are already experienced in performing physical evaluations. However, as patients may not always have ready access to therapists, nurses are more likely to be considered suitable for training initially. Giving nurses the ability to look for and recognize signs of PSS in patients that they may see regularly in community or residential care settings has the potential to increase the number of patients who receive the treatment they need in a timely manner.

The SMD decision tree (23, 24) was developed to help non-specialist nurses and therapists (physiotherapists and occupational therapists) assess the need to refer post-stroke patients to a spasticity specialist. In real-world practice, the final treatment decision would then be made by the specialist, informed by the usual clinical and nonclinical considerations. This decision tree focuses on upper extremities and is used to evaluate three cardinal symptoms of PSS: resistance to passive movement, impaired control of voluntary extensor movements (paresis of the extensor), and stretch-induced pain (25–30). The aim of this study was to assess the practical validity of the decision tree and assess its inter-rater reliability (IRR) by comparing referral decisions between nurses and specialist physicians (the gold standard), and between therapists and specialist physicians.

## 2 Materials and methods

### 2.1 SMD decision tree

The SMD decision tree (23, 24) was developed based on expert consultations, using a three-step Delphi process, patient self-report questionnaires regarding treatment-relevant symptoms, and a focused literature review. Details of these steps are reported elsewhere (23, 31). The decision tree assesses PSS at the elbow, wrist, and fingers as these represent common upper-limb PSS patterns (32–35); although the shoulder (movement) segment is another common site of PSS, this was excluded from the decision tree due to its complexity. The decision tree does not require users to have specialized education in detecting PSS. It evaluates three cardinal symptoms of PSS (resistance to passive movement, impaired control of voluntary extensor movements [paresis of the extensor], and stretch-induced pain) in three segments (elbow, wrist, and fingers) to assess the need for referral to a specialist for further spasticity evaluation and potential BoNT-A treatment. Referral is considered needed if any of the three sites evaluated meet the criteria.

**Figure 1:**
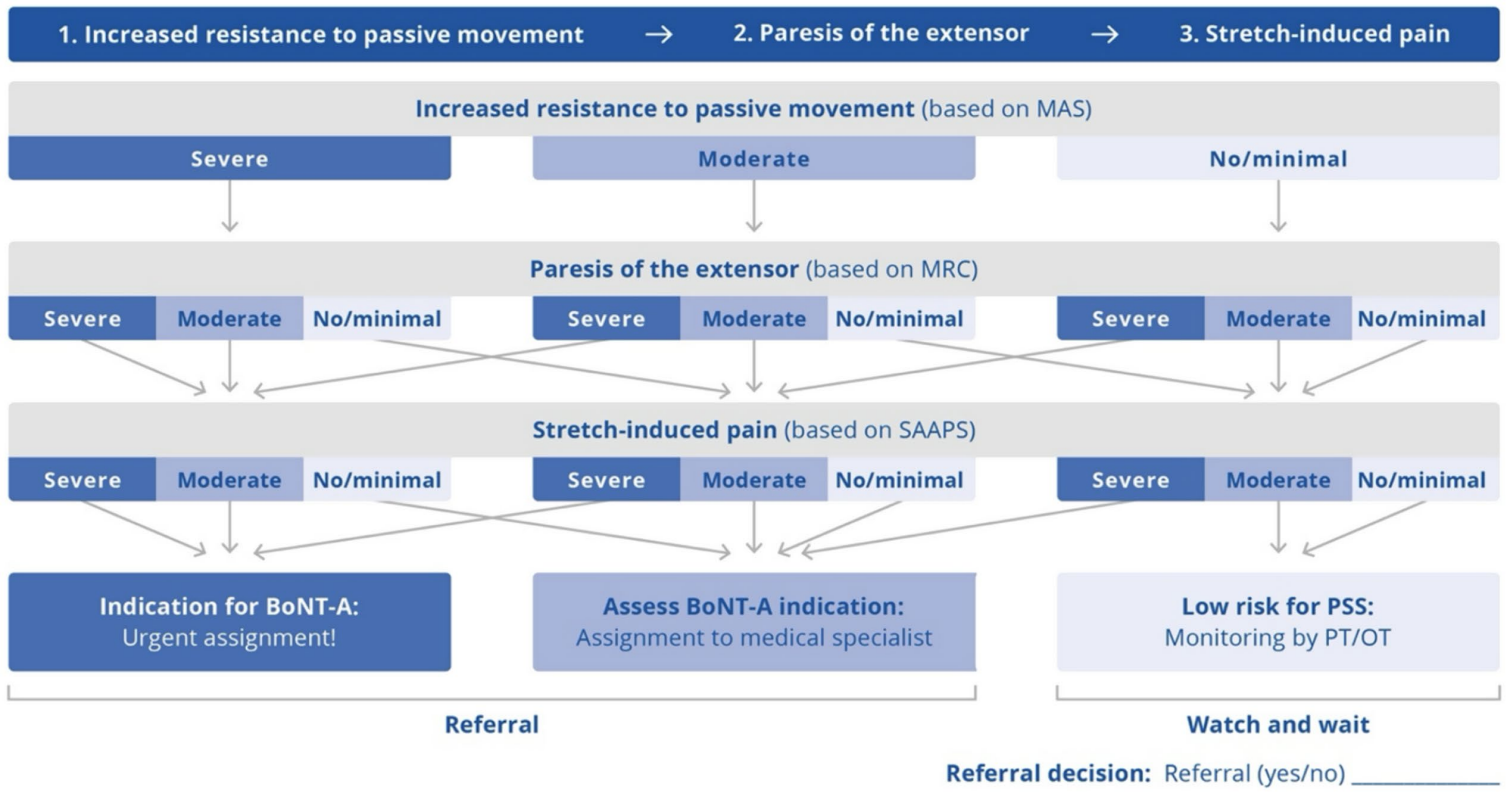
Spastic movement disorders decision tree for the assessment of post-stroke spasticity (PSS) at the elbow, wrist, and fingers. Ratings for each symptom were based on standard scales as follows: Resistance to passive movement: no/minimal = MAS <1+; moderate = MAS 1+; severe = MAS >1+. Paresis of the extensor: no/minimal = modified MRC >3 (standard MRC >2); moderate = modified MRC 3 (standard MRC 2); severe = modified MRC <3 (standard MRC <2). Stretch-induced pain: no/minimal = SAAPS 0; moderate = SAAPS 1; severe = SAAPS >1. BoNT-A, Botulinum neurotoxin type A; MAS, Modified Ashworth Scale; MRC, Medical Research Council; OT, occupational therapist; PT, physiotherapist; SAAPS, Spasticity-Associated Arm Pain Scale.

The decision tree assesses the presence and severity of PSS symptoms based on simplified versions of well-known scales for diagnosing spasticity, namely: the Modified Ashworth Scale (MAS) (28, 29), for the assessment of resistance to passive stretch; the Medical Research Council (MRC) scale (30), for the assessment of voluntary muscle power, an indicator of capability or control of voluntary extensor movements; and the Spasticity-Associated Arm Pain Scale (SAAPS) (27), for the assessment of stretch-induced pain. For the SAAPS, the assessor flexes and extends the joint at a steady, consistent speed throughout the full range of motion. If pain occurs during the very first movement, the test is stopped and recorded as ‘>1’. If pain appears after 2–5 repetitions, the value ‘1’ is documented. If no pain occurs after five repetitions, the value ‘0’ is recorded. The assessments for the MAS, MRC, and SAAPS are performed in a predefined order (Figure 1). In the current study, the numbering of the standard six-point MRC scale, which runs from 0 (no movement) to 5 (segment can be moved with normal force against resistance over the entire range of movement) was modified to align with a 1 to 6 scale as this was felt to be more intuitive to allow ‘3’ to represent the mid-point of the scale (where the modified ‘MRC 3’ corresponded with a standard MRC score of 2 [muscle activation—full range of motion in the segment but not against gravity]). The decision tree was used to rate each symptom as “no/minimal” (based on a MAS score <1+, modified MRC > 3, SAAPS 0 [no pain]), “moderate” (based on MAS 1+, modified MRC 3, SAAPS 1 [pain on repeated movement]), or “severe” (based on MAS > 1+, modified MRC < 3, SAAPS >1 [pain on first bending and stretching movement]) (Supplementary Table S1).

### 2.2 Study design and data collection

The I-REFER study was a single-center, cross-sectional validation study conducted at the Gailtal-Clinic, Hermagor, Austria, chosen for its neurological rehabilitation specialization and the immediate availability of in-house nurses, therapists (physiotherapists and occupational therapists), and specialist physicians.

The study is registered on ClinicalTrials.gov (NCT06381999) and was conducted in accordance with ethical principles that are consistent with the Declaration of Helsinki. The Ethics Committee of the State of Carinthia (Ethikkommission des Landes Kärnten) reviewed and approved the conduct of this study. All patients were thoroughly informed about the study by the principal investigator and signed an informed consent form before participation.

#### 2.2.1 Patients

Patients admitted, predominantly as inpatients, to the Gailtal-Clinic for stroke rehabilitation during the study period (October 2023 to February 2024) were screened for eligibility for this study. The study included patients aged 18–80 years with a first-ever subacute and chronic stroke with ischemia in the middle cerebral artery territory and/or striatocapsular hemorrhage >4 weeks before enrollment; preserved insight and judgment capacity and the ability to give informed consent; and adequate language and task comprehension with an attention span of ≥15 min. Patients with other neurological disorders, BoNT-A treatment <3 months before enrolment, reduced vigilance and attention span of <15 min, requiring adult representation (e.g., the presence of a legally authorized representative), or severe language impairment/motor and sequencing impairments were excluded from the study. Patients were also excluded if they had comorbidities considered to impact passive or active range of motion or upper extremity pain, such as severe arthritis.

#### 2.2.2 Raters

Patients were assessed by three different rater groups: nurses, therapists (physiotherapists and occupational therapists), and specialist physicians. Specialist physicians in our study were physicians experienced in spasticity management and trained in the detection of PSS and treatment with BoNT-A. Nurses and therapists were not previously trained in the detection of PSS but routinely worked with stroke survivors, whereas the physician group comprised two experienced physicians (one rehabilitation specialist and one neurologist with >30 years of experience each) trained in the detection of PSS [including disabling PSS according to the accepted definition (17)] and treatment with BoNT-A, representing the gold standard for assessment.

Nurses and therapists were invited to participate in the study after attending an initial presentation describing the study and its aims. They were trained for 1 h in the use of the decision tree by a physician experienced in SMDs using a standardized approach to test muscle tone and pain via passive movement and to assess the ability of active movements of the affected arm (at the elbow, wrist, and fingers). Training was provided either in person or by a training video (that included a demonstration of a specialist performing an assessment), followed by the opportunity to ask questions.

#### 2.2.3 Assessments

Patients were screened and enrolled in the study before their actual examination day. Data collection took place on four examination days between 2023 (October and November) and 2024 (January and February). However, every patient was assessed by each of the three types of raters (nurses, therapists, and physicians) within 1 examination day and underwent a series of short assessments, guided by the decision tree for nurses and therapists and based on standard diagnostic practice for specialist physicians (clinical examination and the full standard MAS, MRC, and SAAPS scales). All assessments were carried out in the morning and patients were examined in the same position (e.g., upright or seated) regardless of the rater type. To avoid possible effects from stretching the muscles during tests, a break of around 30 min between different rater assessments was mandated and documented. Existing medication regimens for participants were not interrupted but continued as normal and no additional medications were allowed on assessment days.

Patients were assigned to the raters in a partially randomized manner and were blinded to their rater’s professional group. The sequence of assessment was chosen to enable optimal patient flow between assessment stations while ensuring that neither patients nor raters would be able to determine the order of assessments (Supplementary Figure S1). No resting periods or therapy units were allowed between the three assessments. Raters were not permitted to ask patients for their personal details or medical history or to disclose the results of the examination to the patients. On every examination day, two nurses, two therapists, and two physicians were present. Although the nurses and therapists could change across examination days, they remained the same throughout each day. Some patients were randomly selected to be assessed twice by two different nurses on the same examination day to increase data points for IRR analyses between nurses. For patients assessed twice, only one assessment was included in the main analysis: for patients with two complete assessments, one randomly chosen assessment was included, whereas the single complete assessment was included for patients with only one complete assessment. The physician group always consisted of the same two physicians, one of whom worked at the Gailtal-Clinic (principal investigator of the study) and the other as an external medical specialist. Anonymized data were immediately entered into the study-specific electronic case report form by each rater on the day of the examination.

### 2.3 Objectives

The primary objectives of the study were to assess (1) the practical validity of the decision tree used by non-specialized nurses to decide on referral to a medical specialist due to potential PSS, based on a dichotomous outcome of referral/no referral, compared with the gold standard assessment by experienced physicians and (2) the IRR between the two groups, both overall and per segment. The secondary objectives were similar to the primary objectives but compared therapists’ (physiotherapists and occupational therapists) assessments with the gold standard. Exploratory objectives were to assess the IRR (1) between nurses and physicians and (2) between therapists and physicians, in assessments of each neuromuscular symptom (resistance to passive movement, impaired control of voluntary extensor movements [paresis of the extensor], and stretch-induced pain) per segment. The IRR between nurses on the decision for referral was also assessed as a post-hoc analysis.

### 2.4 Statistical analysis

A sample size of 66 patients was planned based on the inter-rater Cohen’s kappa. Using a test power of 80%, an alpha risk of 5%, and assuming a ratio of 70:30 for positive (patient referred to specialist) to negative (patient not referred) results, the calculation assumed an IRR rate of 0.90. Dropouts were not considered due to the cross-sectional design of the study.

Descriptive statistics included means ± standard deviations (SDs) for quantitative variables, and frequencies (*n*) and percentages (%) for categorical variables.

Diagnostic accuracy measures comprising sensitivity (ability of the nurses/therapists to correctly identify patients requiring specialist referral), specificity (ability of the nurses/therapists to correctly identify patients not requiring specialist referral), positive predictive value (PPV; the probability that a patient with a positive result had confirmed PSS) and negative predictive value (NPV; the probability that a patient with a negative result did not have PSS) were calculated to validate the decision tree, as used by nurses and therapists, against the physician gold standard. The IRR and prevalence rate (prevalence of physician assessments that recommended referral to a medical specialist) were also determined. The IRR between nurses and physicians for the dichotomous endpoint (“referral” or “no referral” to a specialist) was estimated using Cohen’s kappa coefficients. Statistical significance was determined using Chi-squared tests.

Sensitivity analyses were conducted for the primary and secondary objectives using a categorical endpoint (low risk for PSS, assess BoNT-A indication, indication for BoNT-A) instead of the dichotomous endpoint (referral or no referral to a specialist). The IRR was assessed using weighted kappa with linear weights, and the strength of association between the judgments was evaluated using contingency coefficients. Chi-squared tests were performed for both types of coefficients.

To evaluate the IRR between nurses on the decision for referral, only patients examined by two nurses were considered. For the other analyses, when patients were assessed by two nurses, only one assessment was selected (with an equal probability of 50% for patients with two complete assessments).

For all IRR analyses, interpretation was as follows: kappa <0.20, slight IRR; 0.21–0.40, fair IRR; 0.41–0.60, moderate IRR; 0.61–0.80, substantial IRR; and >0.80, almost perfect IRR (36).

Statistical analyses were performed using SPSS version 29.0.

## 3 Results

### 3.1 Participants

#### 3.1.1 Patients

Of 72 patients screened, 70 (97.2%) attended the examination day. One patient was excluded because their rating data were incomplete. Of the remaining 69 (95.8%) patients, 47 (68.1%) completed three examinations and 22 (31.9%) completed four examinations.

Patient demographics and clinical characteristics are presented in Table 1. Of the 69 participating patients, 65.2% were male and 94.1% were aged >50 years. Most patients (79.7%) had experienced an ischemic stroke, and 20.3% had experienced a hemorrhagic stroke. At study initiation, more than half of patients (59.4%) were in the late subacute phase [3–6 months post-stroke (37)], 27.6% were in the chronic phase (>6 months post-stroke), and 13.0% were within the early subacute phase (<3 months post-stroke). There were no statistically significant differences between patients according to whether nurses and specialist physicians agreed or disagreed on the need for referral (data not shown).

**Table 1:** Patient demographics and clinical characteristics.

Characteristic, *n* (%)	All patients (*N* = 69)
Sex	
Male	45 (65.2)
Female	24 (34.8)
Age (years)	
18–50	4 (5.9)
51–60	21 (30.4)
61–70	23 (33.3)
71–80	21 (30.4)
Stroke type	
Ischemic stroke	55 (79.7)
Hemorrhagic stroke	14 (20.3)
Stroke stage	
Early subacute phase (<3 months post-stroke)	9 (13.0)
Late subacute phase (3–6 months post-stroke)	41 (59.4)
Chronic phase (>6 months post-stroke)	19 (27.6)
Non-pharmaceutical interventions^a^	
None	8 (11.6)
>10	61 (88.4)
Comorbidities	
None	62 (89.9)
Yes	7 (10.1)
Type of comorbidity^b^	
Chronic pain disorder	3 (4.3)
Cancer	3 (4.3)
Rheumatoid arthritis	1 (1.5)
Concomitant medications	
None	27 (39.1)
Yes	42 (60.1)
Type of concomitant medications	
NSAIDs	11 (15.9)
Opioids	4 (5.8)
Anticonvulsants	11 (15.9)
Antidepressants	31 (44.9)
Muscle relaxants^c^	7 (10.1)
Corticosteroids	2 (2.9)

NSAIDs, non-steroidal anti-inflammatory drugs. ^a^Number of sessions of non-pharmacologic interventions (physiotherapy and/or occupational therapy) in the week before screening. ^b^Information was collected only for comorbidities considered to have a potential effect on the study assessments. ^c^Study protocol did not distinguish between antispastic and antispasmodic agents.

#### 3.1.2 Non-specialized raters (nurses and therapists)

Of the nine raters participating in the study, five (55.6%) were nurses and four (44.4%) were therapists (two occupational therapists and two physiotherapists) (Supplementary Table S2). Most raters were female (77.8%), and ages ranged from 24 to 47 years (mean ± SD age 33.3 ± 8.5 years). The mean ± SD duration of work experience was 9.3 ± 7.8 years. All nine raters worked full-time and four (44.4%) had also worked part-time at some point during their careers. Most raters (77.8%) had taken part in further training, although none were experienced in the detection of PSS.

### 3.2 Validity of the decision tree and inter-rater reliability

#### 3.2.1 Primary objective: decision tree validity and inter-rater reliability between nurses and physicians

**Figure 2:**
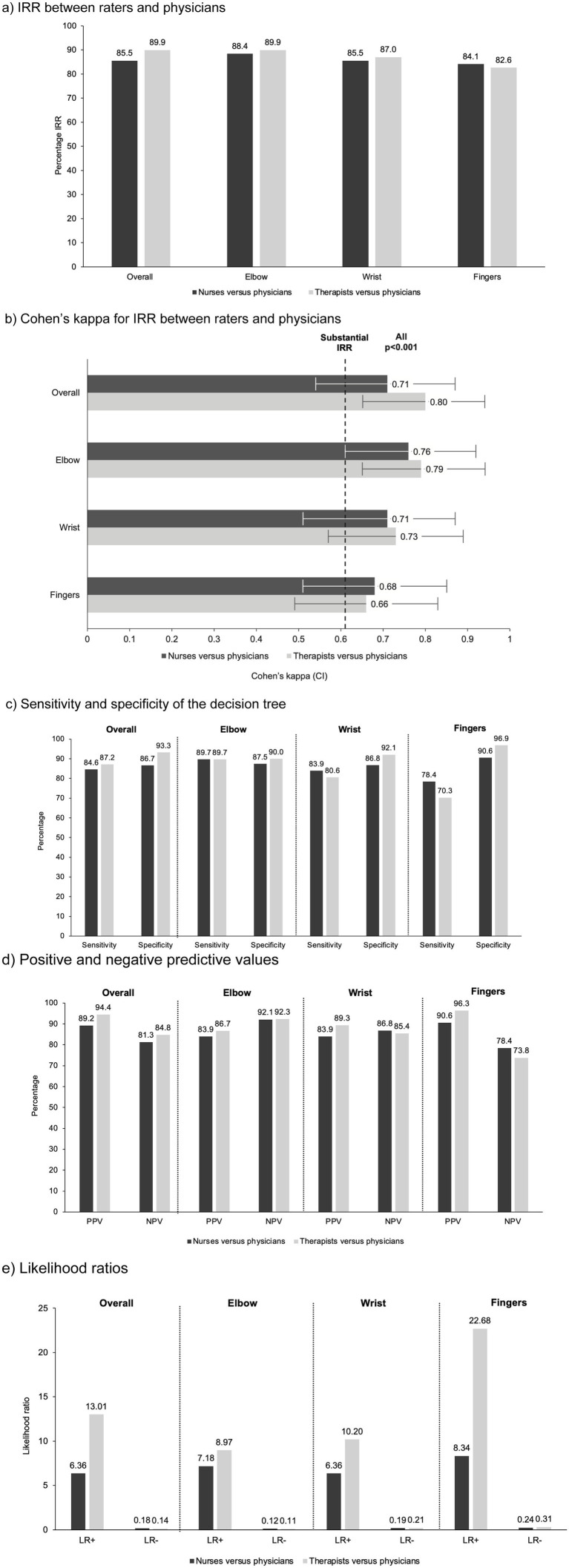
Validity and inter-rater reliability comparing dichotomous results (referral or no referral to a specialist) between raters and physicians. **(a)** IRR between raters and physicians; **(b)** Cohen’s kappa for IRR between raters and physicians; **(c)** Sensitivity and specificity of the decision tree; **(d)** Positive and negative predictive values; **(e)** Likelihood ratios. Prevalence rates for physician assessments that recommended referral to a medical specialist were as follows: overall 56.5%; elbow 42.0%; wrist 44.9%; fingers 53.6%. CI, 95% confidence interval; IRR, inter-rater reliability; LR+, positive likelihood ratio; LR−, negative likelihood ratio; NPV, negative predictive value; PPV, positive predictive value.

Following the decision tree assessments, the percentage of participants who were given positive decisions for specialist referral was 53.6% (by nurses), 52.2% (by therapists) and 56.5% (by physicians) (Supplementary Table S3). IRR between nurses and physicians on the need for referral was high overall (85.5%) and at the elbow (88.4%), wrist (85.5%), and fingers (84.1%; Figure 2a). Cohen’s kappa coefficient showed substantial and statistically significant IRR between nurse and physician assessments at all segments, with an overall coefficient of 0.71 (95% confidence interval [CI] 0.54–0.87; *p* < 0.001; Figure 2b). Nurses’ ability to correctly identify patients requiring specialist referral (sensitivity) and those not requiring specialist referral (specificity) using the decision tree was 84.6 and 86.7%, respectively (Figure 2c). PPVs and NPVs were also high overall (89.2 and 81.3%, respectively) and at all segments evaluated (Figure 2d). Overall, a patient with PSS was 6.36 times (positive likelihood ratio [LR+]) more likely than a patient without PSS to be referred by a nurse to a specialist (positive test result). The probability of a negative test result in patients with PSS was 0.18 times lower than in patients without PSS (negative likelihood ratio [LR-]) (Figure 2e). Results of the examinations per segment and rater group are presented in Supplementary Table S3.

The sensitivity analysis using a categorical endpoint (low risk for PSS, assess BoNT-A indication, indication for BoNT-A) instead of the dichotomous endpoint (referral or no referral to a specialist) indicated an overall IRR between nurses and physicians of 59.4%, with 68.1% IRR for the elbow and fingers and 58.0% for the wrist. Further details are presented in Supplementary Table S4.

#### 3.2.2 Secondary objective: decision tree validity and inter-rater reliability between therapists and physicians

As observed for nurses, there was high IRR between therapists and physicians on the need for specialist referral overall (89.9%) and at the elbow (89.9%), wrist (87.0%), and fingers (82.6%; Figure 2a). Cohen’s kappa coefficient showed substantial and statistically significant IRR between therapists and physicians at all segments, with an overall coefficient of 0.80 (95% CI 0.65–0.94; *p* < 0.001; Figure 2b). Sensitivity and specificity based on therapist ratings using the decision tree were 87.2 and 93.3%, respectively (Figure 2c). PPVs and NPVs were also high overall (94.4 and 84.8%, respectively) and at all segments evaluated (Figure 2d). Patients with PSS were 13.01 times (LR+) more likely than patients without PSS to be referred by a therapist to a specialist, and the probability of a negative test result in patients with PSS was 0.14 times lower than in patients without PSS (LR−) (Figure 2e).

The sensitivity analysis using a categorical endpoint (low risk for PSS, assess BoNT-A indication, indication for BoNT-A) instead of the dichotomous endpoint (referral or no referral to a specialist) indicated an overall IRR between therapists and physicians of 68.3%, with 68.1% IRR for the elbow, 65.2% for fingers, and 59.4% for the wrist (Supplementary Table S4).

#### 3.2.3 Exploratory objectives: inter-rater reliability in assessments of neuromuscular symptoms per (movement) segment

IRR in the assessment of neuromuscular symptoms of resistance to passive movement (based on the MAS), impaired control of voluntary extensor movements (paresis of the extensor; based on the MRC scale), and stretch-induced pain (based on the SAAPS) was assessed as an exploratory endpoint (Table 2).

**Table 2:** Inter-rater reliability in assessments of neuromuscular symptoms per (movement) segment (exploratory objectives).

Assessment	IRR, *n* (%)	Weighted kappa (95% CI)	*p*-value for weighted kappa	Contingency coefficient	*p*-value for coefficient
Exploratory objective 1: nurses versus physicians					
MAS: resistance to passive movement					
Elbow	42 (60.9)	0.54 (0.40–0.69)	<0.001	0.59	<0.001
Wrist	43 (62.3)	0.51 (0.36–0.67)	<0.001	0.55	<0.001
Fingers	44 (63.8)	0.53 (0.36–0.69)	<0.001	0.56	<0.001
MRC: impaired control of voluntary extensor movements					
Elbow	53 (76.8)	0.71 (0.57–0.84)	<0.001	0.65	<0.001
Wrist	51 (73.9)	0.67 (0.54–0.81)	<0.001	0.62	<0.001
Fingers	52 (75.4)	0.68 (0.54–0.82)	<0.001	0.65	<0.001
SAAPS: pain					
Elbow	56 (81.2)	0.25 (0.03–0.48)	0.013	0.47	<0.001
Wrist	57 (82.6)	0.40 (0.15–0.64)	<0.001	0.54	<0.001
Fingers	54 (78.3)	0.38 (0.16–0.59)	<0.001	0.49	<0.001
Exploratory objective 2: therapists versus physicians					
MAS: resistance to passive movement					
Elbow	46 (66.7)	0.56 (0.41–0.72)	<0.001	0.57	<0.001
Wrist	46 (66.7)	0.52 (0.37–0.67)	<0.001	0.57	<0.001
Fingers	52 (75.4)	0.66 (0.52–0.81)	<0.001	0.64	<0.001
MRC: impaired control of voluntary extensor movements					
Elbow	58 (84.1)	0.81 (0.71–0.91)	<0.001	0.71	<0.001
Wrist	49 (71.0)	0.64 (0.50–0.78)	<0.001	0.60	<0.001
Fingers	54 (78.3)	0.73 (0.60–0.86)	<0.001	0.66	<0.001
SAAPS: pain					
Elbow	55 (79.7)	0.33 (0.07–0.60)	<0.001	0.41	0.008
Wrist	57 (82.6)	0.47 (0.21–0.72)	<0.001	0.51	<0.001
Fingers	55 (79.7)	0.48 (0.27–0.69)	<0.001	0.52	<0.001

CI, confidence interval; IRR, inter-rater reliability; MAS, Modified Ashworth Scale; MRC, Medical Research Council; SAAPS, Spasticity-Associated Arm Pain Scale.

The highest IRR between nurses and physicians (exploratory objective 1) was observed for SAAPS (78.3–82.6%), and the lowest was observed for MAS (60.9–63.8%). However, the weighted kappa, which takes into account the degree of disagreement between raters, was highest for the MRC (0.67 [95% CI 0.54–0.81] to 0.71 [95% CI 0.57–0.84]), indicating substantial IRR, and lowest for SAAPS (0.25 [95% CI 0.03–0.48] to 0.40 [95% CI 0.15–0.64]), indicating fair to moderate IRR. The differences between segments were negligible across all scales (Table 2).

Although the IRR between therapists and physicians (exploratory objective 2) on neuromuscular symptoms per segment was higher across all scales than between nurses and physicians, differences persisted for the weighted kappa, which was again generally highest for the MRC (0.64 [95% CI 0.50–0.78] to 0.81 [95% CI 0.71–0.91]; substantial to almost perfect IRR) and lowest for the SAAPS (0.33 [95% CI 0.07–0.60] to 0.48 [95% CI 0.27–0.69]; fair to moderate IRR) (Table 2).

#### 3.2.4 Post-hoc analysis: inter-rater reliability between nurses on the decision for referral

**Figure 3:**
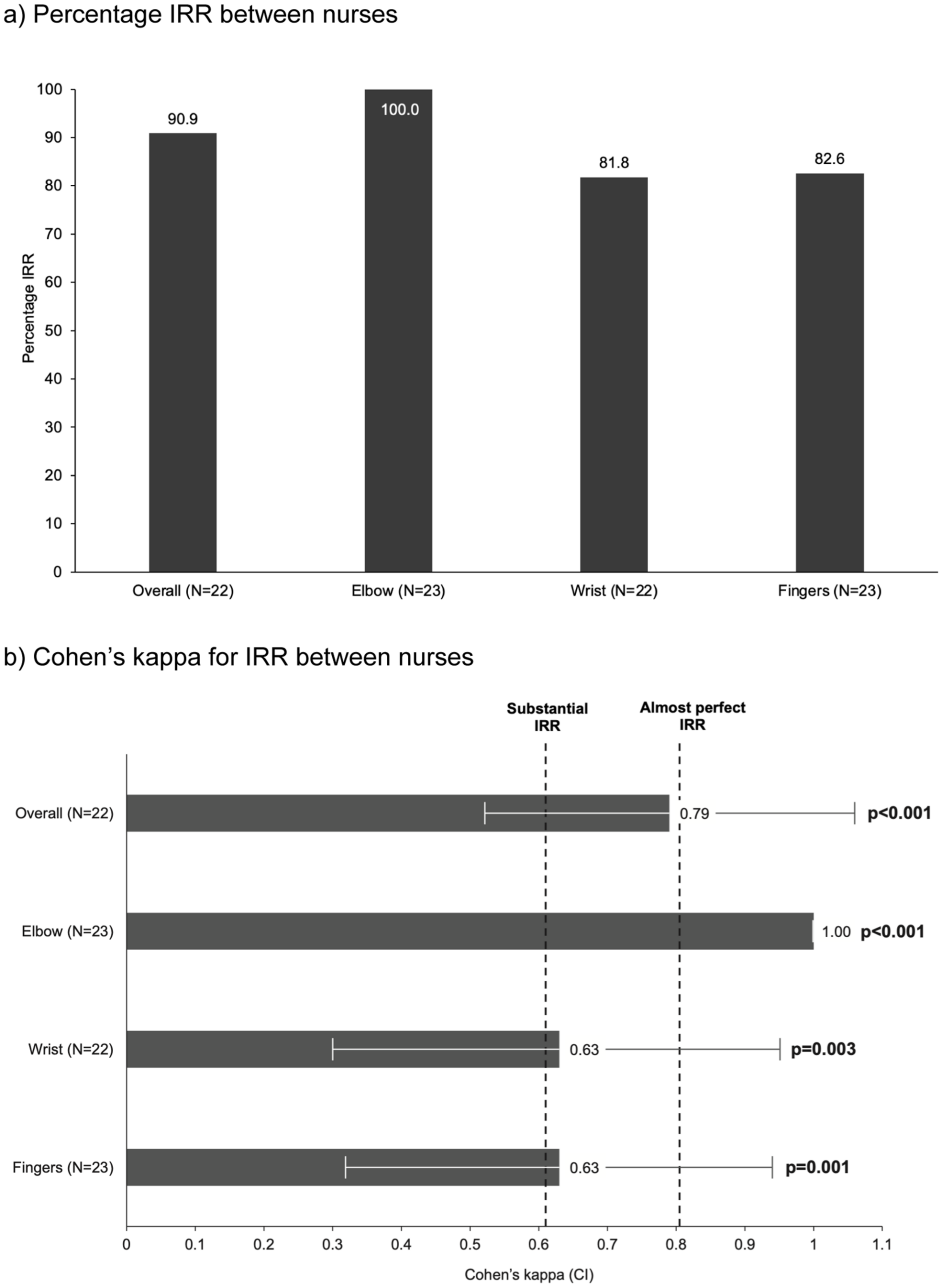
IRR comparing dichotomous results (referral or no referral to a specialist) between nurses (post-hoc analysis). **(a)** Percentage IRR between nurses; **(b)** Cohen’s kappa for IRR between nurses. A total of 24 patients were assessed by two nurses. However, one patient was excluded from these IRR analyses due to an invalid second nurse rating. A second patient was excluded from the overall and wrist analyses as one evaluation of the wrist was incomplete (this patient was only included in the analyses at the elbow and finger). Overall and wrist analyses were therefore based on *N* = 22, and analyses at the elbow and fingers were based on *N* = 23. CI, 95% confidence interval; IRR, inter-rater reliability.

For patients assessed by two nurses, the proportion of overall IRR between nurses’ assessments was 90.9%, with substantial IRR indicated by a Cohen’s kappa of 0.79 (95% CI 0.52–1.0; *p* < 0.001) (Figure 3). The IRR was highest for the elbow (100%), followed by the fingers (82.6%) and the wrist (81.8%). Perfect IRR was achieved for assessments on the elbow, with those on the wrist and fingers showing substantial IRR.

## 4 Discussion

This study supports the validity of the SMD decision tree when used by nurses and therapists to identify patients with PSS in the upper extremities, compared with “gold standard” assessment by specialist physicians. PSS is currently underdiagnosed and undertreated (6, 9, 21, 22). The decision tree offers a simple but extremely useful tool to enable and empower non-specialized nurses and therapists to help improve patient outcomes by increasing the rates of PSS diagnosis and early treatment opportunities.

The overall IRR between nurse and therapist raters and physicians was substantial when determining the need for referral to a specialist. Therapists showed slightly higher levels of concordance with physicians than with nurses, likely because therapists are more frequently involved in evaluating and treating patients for their spasticity. The decision tree also showed high sensitivity, specificity, and positive and negative predictive values; these were accompanied by high LR+ values (showing a low risk of a false-positive result) and low LR− values (showing a low risk of a false-negative result). Sensitivity analyses based on the categorical endpoint (low risk for PSS, assess BoNT-A indication, indication for BoNT-A) instead of the dichotomous endpoint (referral or no referral to a specialist) showed similar trends, although IRR versus physicians was lower for both nurses and therapists than with the dichotomous results. A possible explanation for this is that physicians were more inclined to recommend “indication for BoNT-A,” whereas nurses and therapists more frequently recommended “assess BoNT-A indication.” This cautious approach by nurses and therapists may be expected, as they do not have authority in current clinical practice in most European countries to make decisions about the necessity of BoNT-A treatment. Previous feedback from nurses included feeling insecure and concerned about doing something wrong due to lack of experience in structured PSS assessment (38). Therefore, the sensitivity analyses provide theoretical insights rather than practical implications. Although the main objectives of this study were assessed based on a simplified dichotomous outcome of referral/no referral to a specialist, the final decision tree offers the option to prioritize patients with a potential “indication for BoNT-A” for urgent referral.

Findings were also similar based on assessments at the (movement) segment level. The slightly lower concordance for the finger could be because the finger was the smallest segment assessed, making it more difficult to discriminate between levels of impairment than with the larger segments. However, differences were not considered large enough to be clinically relevant (i.e., to affect the referral decision) and were not supported by sensitivity analyses, where IRR was lowest for the wrist.

The overall ratings of neuromuscular symptoms by nurses and therapists compared with specialist physicians showed a higher IRR for motor control/active movement (substantial IRR) than for resistance to passive movement and pain (moderate to fair IRR), which may warrant further study. Potential reasons for this may include that active movement is based on learned activity while passive movement is not consciously learned, possibly making impaired active movements easier to recognize. Motor control/active movement is also assessed by observation, whereas assessment of resistance to passive movement and pain may require more experience. Again, differences between assessments at the segment level were negligible.

The decision tree provides a potential opportunity for the early detection of PSS by non-specialist assessment and differs from other spasticity screening tools in important aspects. In 2017, Zorowitz et al. (39) developed a 13-item patient-reported screening tool with the aim of identifying patients affected by spasticity who require treatment, although the cause of spasticity was not limited to stroke. The nature of the patient questionnaire means that no physical examination is carried out to assess potential spasticity. Although the Zorowitz et al. (39) questionnaire was developed by an expert Delphi panel and patient interviews, it has not been fully validated or assessed for use by non-specialist practitioners; there is also no specified ‘cut-off point’ at which patients would be referred for further assessment. Although pain is mentioned in one question of the Zorowitz et al. tool, our SMD decision tree is the first screening tool to feature pain as one of three cardinal symptoms of PSS. Although resistance to passive movement, followed by active movement impairment, remain the main drivers for specialist referral, pain is an important consideration as it is another common treatment goal for patients with spasticity (40).

The “simple bedside screening tool for spasticity referral” is another tool that facilitates decision-making about referral to a medical specialist based on a brief physical examination and provides a flowchart for guidance on when to refer to a spasticity specialist (41). The three-question flowchart is intended to be used by primary care physicians and nurse practitioners not experienced in the detection of spasticity. Although the bedside screening tool showed high sensitivity and moderate specificity, the SMD decision tree demonstrated both high sensitivity and high specificity. A particular strength of the SMD decision tree is that it allows multiple chances to determine a positive referral by including a predefined flow of decision points through three separate sites. This feature may contribute to its high sensitivity (identification of true positives) but, importantly, does not compromise its specificity (i.e., it had a lower rate of false positives than the bedside screening tool). As may be expected for a bedside tool, its assessment is broader than our decision tree and assesses both upper and lower limbs based on two questions about abnormal posture and perceived resistance of the affected limbs but does not cover spasticity-associated pain (41). The SMD decision tree is more specialized, based on three validated scales relevant for PSS (MAS, MRC, and SAAPS); this requires more time but allows assessment of the three hallmark signs of disabling PSS. The scales were simplified to fewer categories to avoid creating a tool that was overly complex to nonspecialists. The positive findings of this study demonstrate that the SMD decision tree was simple to use and 1 h of training was adequate.

Another available tool is the “PSS risk classification tool,” which outlines the next steps according to the level of urgency for referral (urgent referral, routine referral, periodic monitoring) based on PSS risk factors (42). However, this tool has not been validated, and no specific guidelines have been published. Moreover, the intended professional audience for this tool is not clearly defined.

This study shows strong evidence for the use of the SMD decision tree to identify patients with potential harmful PSS by non-specialist raters. The study was performed in a group of patients with particular stroke characteristics (e.g., ischemia in the middle cerebral artery territory) and spasticity patterns (i.e., elbow, wrist and/or finger spasticity) in order to create a homogeneous sample. However, as the decision tree is based on well-established scales (MAS, MRC, and SAAPS), it is likely that its use could be broadened to benefit the diagnosis of spasticity due to any type of stroke, as well as due to non-stroke-related etiologies, such as traumatic brain injury or adult cerebral palsy, although further work would be needed to confirm this. Further, given this positive proof-of-concept in PSS of the upper extremities, feasibility of a similar referral decision tree using lower limbs and extremities may be explored. This study was performed in a specialized clinic, and further research could examine the potential for implementation of the tool into other standard care environments where nurses may be present but specialized doctors are rare, such as private home visits, residential homes, and primary care. This could enable pre-selection of patients with PSS by nurses for specialist follow-up and potential BoNT-A injection. The tool may also be particularly useful for rural communities, and further research could explore its potential for use in developing countries where access to specialized services may be particularly limited.

Although this study provides positive evidence for the validity of the decision tree, some limitations need to be acknowledged. As this was a single-center study, the diversity of the raters may be limited, potentially affecting the generalizability of the results. The nine nurses and therapists who participated as non-specialist raters in the study frequently interacted with stroke survivors during their usual course of work at a specialized rehabilitation center; consequently, these raters had more experience with PSS and may therefore be more likely to detect potentially disabling spasticity than their counterparts in less specialized environments. However, it should be noted that none of the raters had specific education in spasticity, and some had relatively little professional experience (range 2–25 years). They also received only 1 h of training on the use of the decision tree. This suggests that nurses and therapists with fewer interactions with stroke survivors can also be effectively trained to use the decision tree in a time- and resource-efficient manner, and work is ongoing to develop and evaluate appropriate training materials (25) to potentially enable patients to be referred to specialists (and receive appropriate timely treatment), even when they are not initially assessed in specialist spasticity centers. The high IRR among nurses’ ratings also indicates that the varying characteristics of the raters do not significantly influence the results of assessment using the decision tree. Following the completion of the current study, it was noted that the use of a modified 1–6 scale to represent the six-point MRC scale in the decision tree (rather than the standard 0–5 MRC scale), that is, where the modified “MRC 3″ corresponded with a standard MRC score of 2 (muscle activation against gravity), may cause confusion. As nurses and therapists are generally unlikely to be familiar with the numbering of the standard MAS, MRC scale, and SAAPS, this is unlikely to have affected our results. However, the decision tree is now based solely on options of “no/minimal,” “moderate,” or “severe” for each of the three main PSS symptoms (Figure 1) (24); using this approach, scores of modified “MRC 3″ or lower (standard MRC scale score 2 or lower) would be classed as “moderate” or “severe” and therefore result in referral. Finally, the double-blinding design was a strength of the study; however, a patient may have known their rater and vice versa, which may be a limitation.

Despite these limitations, our findings show that the decision tree has the potential to improve the care of patients with PSS. Its ease of use allows rapid patient assessment, supporting its introduction as a routine tool that can be readily integrated into the day-to-day work of nurses and therapists. Further analyses are needed to examine the full impact of the SMD decision tree on number of referrals and to explore whether routine use of the decision tree translates into the expected increase in early specialist assessment and timely access to appropriate BoNT-A, ultimately improving the lives of people with PSS and their families and reducing the associated healthcare and economic burdens.

## 5 Conclusion

The SMD decision tree showed high validity and reliability for effectively helping nurses and therapists to recognize potential PSS in the upper extremities and decide on referral to a specialist for timely diagnosis and potential BoNT-A treatment. Empowering nurses and therapists to screen for potentially disabling PSS could help to alleviate the burden on specialist physicians and expedite patient access to appropriate care as part of a multimodal approach. Given the robustness of the tool and the potential positive impact on clinical practice and patient health, it is recommended that this tool be introduced into standard clinical practice and its potential for broader use in community settings, where access to specialist is limited and nurses may play a vital role in early detection of PSS, be examined. Further work is also recommended to examine the potential for use of the decision tree to aid in the diagnosis of spasticity due to non-stroke-related etiologies, such as traumatic brain injury or adult cerebral palsy.

## Data Availability

The original contributions presented in the study are included in the article/Supplementary material; further inquiries can be directed to the corresponding author.
