# Supplementary material for: Early recognition of post-stroke spasticity: the I-REFER study
Source: Front Neurol. 2026 May 13;17:1735793. doi: 10.3389/fneur.2026.1735793 (PMC13215129; doi:10.3389/fneur.2026.1735793)

## Supplementary Table S1 Summary of Decision Tree Ratings per Symptom

| **Rating^a^** | **Symptom** | | |
| --- | --- | --- | --- |
|  | **Resistance to passive movement** | **Impaired control of voluntary extensor movements (paresis of the extensor)** | **Stretch-induced pain** |
|  | **Assessment score and description^b^** | | |
|  | **MAS** | **Modified MRC** | **SAAPS** |
| No/minimal | <1+  No or minimal increase in muscle tone with slight resistance (catch) at the end of the entire movement path (less than half) when stretching the target joints | >3  Active movement during the entire range of motion against resistance/ gravity | 0  No pain with 5 stretching and bending movements |
| Moderate | 1+  Slight increase in muscle tone with slight resistance (catch) when stretching the target joints to at least half of the range of motion | 3  Active movement possible during range of motion, but not against resistance/ gravity | 1  Pain with repeated movements (maximum 5 repetitions) |
| Severe | >1+  Significant increase in muscle tone over the entire range of motion when stretching the target joints | <3  No active movement possible during the entire range of motion | >1  Pain on first bending and stretching movement |

^a^Rating indicates presence/severity of symptom; one rating was made for each of the three joints (elbow, wrist, finger).

^b^Original versions of the assessments were simplified for use in the decision tree.

## Supplementary Table S2 Characteristics and Work Experience of Non-Specialized Raters (Nurses and Therapists)

| **Characteristic** | **All non-specialized raters**  **(N=9)** |
| --- | --- |
| Age, years | 33.3 ± 8.5 (24–47) |
| Sex |  |
| Female | 7 (77.8) |
| Male | 2 (22.2) |
| Type of education/profession |  |
| Nurse | 5 (55.6) |
| Therapist | 4 (44.4) |
| Occupational therapist | 2 (22.2) |
| Physiotherapist | 2 (22.2) |
| Duration of education |  |
| 3 years | 8 (88.9) |
| 5 years | 1 (11.1) |
| Final year of education |  |
| 1998 | 1 (11.1) |
| 2006 | 1 (11.1) |
| 2012 | 1 (11.1) |
| 2015 | 1 (11.1) |
| 2016 | 1 (11.1) |
| 2018 | 1 (11.1) |
| 2021 | 3 (33.3) |
| Duration of work experience, years | 9.3 ± 7.8 (2–25) |
| Working model |  |
| Full-time | 9 (100.0) |
| Part-time | 4 (44.4) |
| Duration of full-time work, years | 5.1 ± 2.4 (2–9) |
| Duration of part-time work, years | 4.2 ± 6.1 (3–16) |
| Receipt of further education^a^ |  |
| Yes | 7 (77.8) |
| No | 2 (22.2) |
| Duration of further education^a^, hours | 9.2 ± 6.0 (1–19) |

Data are presented as mean ± standard deviation (range) or n (%)

^a^Further education beyond standard training for nurses/therapists

## Supplementary Table S3 Results of Each Scale and Decision for Referral per (Movement) Segment and Rater Group (N=69 Patients)

| **Characteristic** | **Nurse** | | | **Therapist** | | | **Physician** | | |
| --- | --- | --- | --- | --- | --- | --- | --- | --- | --- |
|  | **Elbow** | **Wrist** | **Fingers** | **Elbow** | **Wrist** | **Fingers** | **Elbow** | **Wrist** | **Fingers** |
| **MAS** |  |  |  |  |  |  |  |  |  |
| >1+ | 21 (30.4) | 16 (23.2) | 24 (34.8) | 14 (20.3) | 7 (10.1) | 12 (17.4) | 16 (23.2) | 16 (23.2) | 21 (30.4) |
| 1+ | 23 (33.3) | 22 (31.9) | 14 (20.3) | 15 (21.7) | 21 (30.4) | 15 (21.7) | 14 (20.3) | 15 (21.7) | 7 (10.1) |
| <1+ | 25 (36.3) | 31 (44.9) | 31 (44.9) | 40 (58.0) | 41 (59.4) | 42 (60.9) | 39 (56.5) | 38 (55.1) | 41 (59.5) |
| **MRC^a^** |  |  |  |  |  |  |  |  |  |
| <3 | 14 (20.3) | 15 (21.7) | 18 (26.1) | 14 (20.3) | 15 (21.7) | 20 (29.0) | 18 (26.1) | 22 (31.9) | 27 (39.1) |
| 3 | 11 (15.9) | 15 (21.7) | 11 (15.9) | 13 (18.8) | 15 (21.7) | 10 (14.4) | 12 (17.4) | 7 (10.1) | 10 (14.5) |
| >3 | 44 (63.8) | 39 (56.5) | 40 (58.0) | 42 (60.9) | 39 (56.6) | 39 (56.6) | 39 (56.5) | 40 (58.0) | 32 (46.4) |
| **SAAPS** |  |  |  |  |  |  |  |  |  |
| >1 | 3 (4.3) | 4 (5.8) | 5 (7.2) | 9 (13.1) | 5 (7.2) | 6 (8.7) | 3 (4.3) | 4 (5.8) | 7 (10.1) |
| 1 | 7 (10.1) | 5 (7.2) | 7 (10.1) | 5 (7.2) | 7 (10.1) | 9 (13.0) | 5 (7.2) | 5 (7.2) | 7 (10.1) |
| 0 | 59 (85.6) | 60 (87.0) | 57 (82.7) | 55 (79.7) | 57 (82.7) | 54 (78.3) | 61 (88.5) | 60 (87.0) | 55 (79.8) |
| **Suggestion for referral** | | | | | | | | | |
| *By joint* | | | | | | |  |  |  |
| No referral | 38 (55.1) | 38 (55.1) | 37 (53.6) | 39 (56.5) | 41 (59.4) | 42 (60.9) | 40 (58.0) | 38 (55.1) | 32 (46.4) |
| Referral | 31 (44.9) | 31 (44.9) | 32 (46.4) | 30 (43.5) | 28 (40.6) | 27 (39.1) | 29 (42.0) | 31 (44.9) | 37 (53.6) |
| *Overall* | | | | | | | | | |
| No referral | 32 (46.4) | | | 33 (47.8) | | | 30 (43.5) | | |
| Referral | 37 (53.6) | | | 36 (52.2) | | | 39 (56.5) | | |

Data are presented as n (%)

MAS, Modified Ashworth Scale; MRC, Medical Research Council; SAAPS, Spasticity-Associated Arm Pain Scale

^a^In the current study, the numbering of the standard six-point MRC scale, which runs from 0 (no movement) to 5 (segment can be moved with normal force against resistance over the entire range of movement), was modified to align with a 1–6 scale to allow a modified score of “3” to represent the mid-point of the scale (where modified “MRC 3” corresponded with a standard MRC score of 2 [muscle activation – full range of motion in the segment but not against gravity]). The decision tree was used to rate each symptom as “no/minimal” (based on a MAS score <1+, modified MRC >3, SAAPS 0), “moderate” (based on MAS 1+, modified MRC 3, SAAPS 1), or “severe” (based on MAS >1+, modified MRC <3, SAAPS >1; [Figure 1]).

Shading reflects trends in the results, with darker shades representing greater proportions and the lighter shades representing smaller proportions. No tests were conducted to assess statistical differences.

## Supplementary Table S4 Sensitivity Analysis of Inter-Rater Reliability Based on a Categorial Endpoint

|  | **IRR, n (%)** | **Weighted kappa (95% CI)** | **p-Value for weighted kappa** | **Contingency coefficient** | **p-Value for coefficient** |
| --- | --- | --- | --- | --- | --- |
| **Nurses versus physicians** | | | | | |
| **Overall** | **41 (59.4)** | **0.49 (0.34–0.64)** | **<0.001** | **0.59** | **<0.001** |
| Elbow | 47 (68.1) | 0.52 (0.37–0.67) | <0.001 | 0.61 | <0.001 |
| Wrist | 40 (58.0) | 0.43 (0.31–0.54) | <0.001 | 0.65 | <0.001 |
| Fingers | 47 (68.1) | 0.56 (0.41–0.71) | <0.001 | 0.62 | <0.001 |
| **Therapists versus physicians** | | | | | |
| **Overall** | **44 (68.3)** | **0.58 (0.44–0.71)** | **<0.001** | **0.64** | **<0.001** |
| Elbow | 47 (68.1) | 0.55 (0.42–0.69) | <0.001 | 0.63 | <0.001 |
| Wrist | 41 (59.4) | 0.47 (0.36–0.58) | <0.001 | 0.66 | <0.001 |
| Fingers | 45 (65.2) | 0.52 (0.38–0.67) | <0.001 | 0.62 | <0.001 |

BoNT-A, Botulinum neurotoxin type A; CI, confidence interval; IRR, inter-rater reliability; PSS, post-stroke spasticity

The sensitivity analysis was based on a categorical endpoint (low risk for PSS, assess BoNT-A indication, indication for BoNT-A) instead of the dichotomous endpoint (referral or no referral to a specialist)

***Supplementary Figure S1 Group Allocation and Patient Blinding***


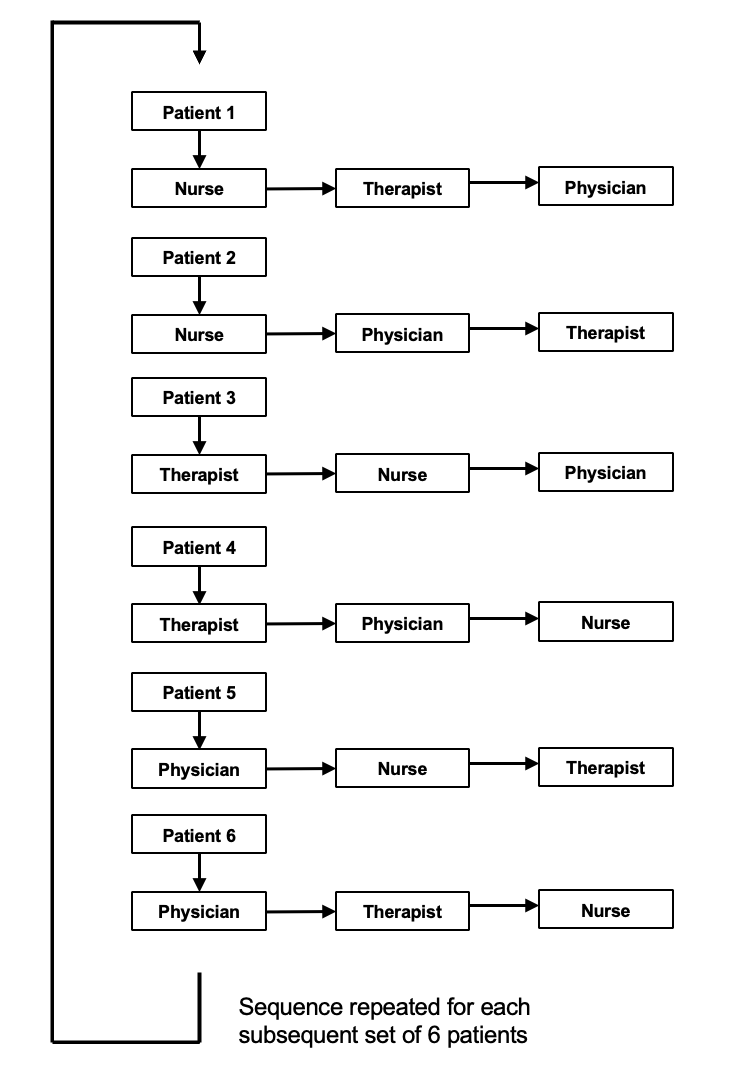

Supplement: Supplementary file 1 [file Supplementary_File_1.docx]
